# Genetic Diversity and Population Structure of *Cannabis* Based on the Genome-Wide Development of Simple Sequence Repeat Markers

**DOI:** 10.3389/fgene.2020.00958

**Published:** 2020-09-11

**Authors:** Jiangjiang Zhang, Jiangtao Yan, Siqi Huang, Gen Pan, Li Chang, Jianjun Li, Chao Zhang, Huijuan Tang, Anguo Chen, Dingxiang Peng, Ashok Biswas, Cuiping Zhang, Lining Zhao, Defang Li

**Affiliations:** ^1^Research Team of Genetic Modification of Annual Bast Fiber Crops, Institute of Bast Fiber Crops, Chinese Academy of Agricultural Sciences, Changsha, China; ^2^Key Laboratory of Biological and Processing for Bast Fiber Crops, Ministry of Agriculture, Changsha, China; ^3^MOA Key Laboratory of Crop Ecophysiology and Farming System in the Middle Reaches of the Yangtze River, College of Plant Science and Technology, Huazhong Agricultural University, Wuhan, China

**Keywords:** *Cannabis*, genetic diversity, population structure, simple sequence repeat, cluster analysis

## Abstract

*Cannabis* has been used as a source of nutrition, medicine, and fiber. However, lack of genomic simple sequence repeat (SSR) markers had limited the genetic research on *Cannabis* species. In the present study, 92,409 motifs were identified, and 63,707 complementary SSR primer pairs were developed. The most abundant SSR motifs had six repeat units (36.60%). The most abundant type of motif was dinucleotides (70.90%), followed by trinucleotides, tetranucleotides, and pentanucleotides. We randomly selected 80 pairs of genomic SSR markers, of which 69 (86.25%) were amplified successfully; 59 (73.75%) of these were polymorphic. Genetic diversity and population structure were estimated using the 59 (72 loci) validated polymorphic SSRs and three phenotypic markers. Three hundred ten alleles were identified, and the major allele frequency ranged from 0.26 to 0.85 (average: 0.56), Nei’s genetic diversity ranged from 0.28 to 0.82 (average: 0.56), and the expected heterozygosity ranged from 0.28 to 0.81 (average: 0.56). The polymorphism information content ranged from 0.25 to 0.79 (average: 0.50), the observed number of alleles ranged from 2 to 8 (average: 4.13), and the effective number of alleles ranged from 0.28 to 0.81 (average: 0.5). The *Cannabis* population did not show mutation-drift equilibrium following analysis via the infinite allele model. A cluster analysis was performed using the unweighted pair group method using arithmetic means based on genetic distances. Population structure analysis was used to divide the germplasms into two subgroups. These results provide guidance for the molecular breeding and further investigation of *Cannabi*s.

## Introduction

*Cannabis* is an erect annual herb that belongs to the family Cannabaceae. Its cultivation, as a source of fiber, was first documented in China and, subsequently, spread throughout the world (Li, 1973; Small and Cronquist, 1976). As one of the oldest plants, *Cannabis* has been used medicinally for more than 10,000 years in China, and this is documented by Emperor Shen Neng (Schultes et al., 1974); the plant has regained interest and popularity for its potential therapeutic effects. There are many standards to classify *Cannabis* species. From a utilitarian perspective, *Cannabis* is classified into four types, namely wild, fiber, oilseed, and psychoactive types. Taxonomically, *Cannabis* is recognized as hemp and marijuana. Based on the degree of domestication, *Cannabis* is classified as wild, domesticated, and intermediate types (Chandra et al., 2017). Previous studies demonstrate substantial genetic diversity between marijuana and hemp lines. There are considerable morphological variations between wild and cultivated *Cannabis* types; furthermore, *Cannabis sativa* is less variable and relatively more homogenous than *Cannabis indica* (Lynch et al., 2016). Tools to exploit and dissect the *Cannabis* genome have been investigated for several decades. Molecular markers have been utilized to the identify sexual phenotypes and chemotype-determining factors and elucidate the genetic diversity of *Cannabis* species (Mandolino and Carboni, 2004).

Cannabidiol (CBD) and tetrahydrocannabinol (THC) are components in *Cannabis*, and they have unique effects on mental health (Pate, 1994; Schluttenhofer and Yuan, 2019). *Cannabis* has become popular in clinical research owing to its nonintoxicating effects. It is also used to treat epilepsy and addiction and to control pain (Teitelbaum, 2019). THC is known for its hallucinogenic qualities, and recently, it has been shown to be effective in the treatment of Alzheimer’s disease (Cao et al., 2014) and glaucoma (Flach et al., 2002). Cannabigerolic acid (CBDA) is the precursor substance to CBD; when heating tissues *in vitro*, CBDA translates into CBD.

Genetic diversity is usually estimated using DNA sequences (polymorphisms among varieties) and cytological and morphological markers. However, morphological characteristics are often influenced by the environment. Therefore, molecular markers are relatively more stable and popular than morphological markers (Nadeem et al., 2018). Genetic diversity can be used to assess the evolution and conservation of varieties (Ellegren and Galtier, 2016). The events of inbreeding and evolution might alter allele frequency and reduce genetic diversity (Hufbauer et al., 2004). Thus, it is vital to accurately estimate the correlation among different germplasm resources to ensure high-efficiency utilization and management and to maintain adequate genetic variability for breeding several plant varieties (Nderu et al., 2019). Genetic diversity and population structure analysis have been used for the examination of various plant species. An analysis of 1151 ramie germplasms using SSR and phenotypic markers reveals that the genetic diversity of wild germplasms is higher than that of domesticated germplasms. Meanwhile, the population is clustered into two subpopulations (Feng et al., 2018). An analysis of genetic diversity of 50 populations of confectionery sunflower in Iran reveals that these can be subdivided into two subpopulations (Jannatdoust et al., 2016). Further, a population structure analysis of 109 Brazilian common bean accessions shows that these accessions can be divided into two distinct subpopulations (Valentini et al., 2018).

The genetic diversity of different germplasm resources of *Cannabis* has been estimated using molecular markers as well. A single-hexanucleotide short tandem repeat (STR) named NMI101 was employed to analyze the distribution of 93 processed seeds (Shirley et al., 2013). An analysis of 81 marijuana and 43 hemp samples using single-nucleotide polymorphisms (SNPs) revealed that marijuana and hemp were significantly different at the genome-wide level and that hemp was genetically more similar to the *Cannabis indica* type than the *Cannabis sativa* type (Sawler et al., 2015). Expressed sequence tag SSRs (EST-SSRs) were used to assess the genetic diversity of 115 hemp germplasm resources, and they were divided into four groups (Gao et al., 2014). The molecular markers of SSRs are proven to be highly polymorphic, and genomic SSRs are more polymorphic and stable than EST-SSRs (Soler et al., 2017). Several studies focus on the classification of *Cannabis*, including their population structure and genealogical classification, but the relationship between Chinese and foreign strains is also unclear. For the identification of relatedness between strains, more accurate molecular markers are required to analyze their population diversity.

The aim of our study is to develop abundant markers for *Cannabis* species to increase the number of *Cannabis*-related molecular markers available. Here, we identify SSR motifs and developed genomic SSR markers and simultaneously select and validate polymorphisms in these markers to estimate the population structure and genetic diversity of *Cannabis* germplasm resources using three phenotypic markers.

## Materials and Methods

### Experimental Materials

A total of 199 *Cannabis* germplasm resources were collected from 20 regions across China—including Chongqing (1), Guangxi (1), Yunnan (28), Liaoning (3), Guizhou (1), Henan (9), Hebei (1), Shandong (7), Shanxi (7), Heilongjiang (30), Jilin (7), Zhejiang (3), Jiangsu (2), Anhui (4), Xinjiang (3), Gansu (11), Ningxia (3), Qinghai (1), Shaanxi (6), and Inner Mongolia (7)—Germany (1), Hungary (1), Poland (20), Ukraine (9), Kazakhstan (2), Russia (23), Uzbekistan (1), the United States (1), Lithuania (1), and Bosnia and Herzegovina (1). Testing materials were provided by the Research Team of Genetic Modification of Annual Bast Fiber Crops, Institute of Bast Fiber Crops, Chinese Academy of Agricultural Sciences.

In April 2018, all 199 *Cannabis* germplasm resources were sown in Changsha farm, offered by the Institute of Bast Fiber Crops, Chinese Academy of Agricultural Sciences. In May 2018, fresh top leaves were collected from each female accession and placed in liquid nitrogen canisters, transported to the laboratory, and stored at −80°C until genomic DNA extraction.

### Development of SSRs on Whole Genome-Wide Scale

Genomic SSRs were developed based on the whole-genome sequence of *Cannabis sativa* (Laverty et al., 2019). MIcroSAtellite^1^ was used to identify SSR motifs (Beier et al., 2017). The motifs were constrained in length to 2–6 bp, which corresponded to dinucleotides (di-), trinucleotides (tri-), tetranucleotides (tetra-), pentanucleotides (penta-), and hexanucleotides (hexa-), respectively. The SSR markers were developed using Primer3 online (Whitehead Institute, Cambridge, MA, United States). The maximum scores were selected for the SSR locus. The general primer picking conditions were as follows: primer size, 18–27 bp with an optimal length of 24 bp; primer melting temperature (*Tm*), 57–63°C with an optimum temperature of 60°C; product size, 100–200 bp with an optimum length of 150 bp; and primer GC content, 40–60% with an optimum content of 50%.

### Validation of SSRs

Genomic DNA was extracted from the leaves using a rapid DNA extraction kit (DP305; Tiangen Biotech Co. Ltd., Beijing, China). The quality and purity of the extracted DNA were assessed by 1% agarose gel electrophoresis. The SSR-PCR reaction system was performed using MastMIX (KT201; Tiangen Biotech Co. Ltd., Beijing, China). The mix contained 250 μM dNTPs, 10 mM Tris-HCl, 50 mM KCl, and 1.5 mM MgCl_2_. Each total volume of the SSR-PCR mixture was 10 μL, comprising 6.5 μL of MastMIX, 2 μL of double distilled H_2_O, and 30 ng of template DNA. The PCR amplification was carried out using a Bio-Rad (Hercules, CA, United States) thermal cycler under the following cycling conditions: initial denaturation at 94°C for 5 min, 37 cycles at 94°C for 30 s, 50°C–60°C for 30 s, 72°C for 40 s, and a final extension at 72°C for 10 min. The genotype of each accession was investigated by polyacrylamide gel electrophoresis (PAGE) at 150 V for 2.5 h. The PAGE gel consisted of 10% acrylamide, 10% TBE, and 1.5% ammonium persulfate. The bands were stained with 2 mg/mL silver nitrate for 10 min.

Eight pairs of SSR markers were randomly selected from each chromosome; three pairs of dinucleotide SSRs, two pairs of trinucleotide SSRs, and one pair each of tetra-, penta-, and hexa- SSRs. A total of 80 SSR markers were selected to validate the polymorphism and specificity of 12 different *Cannabis* varieties.

### Morphological Characterization

The contents of CBD, THC, and CBDA in the leaves at the flowering stage were determined by HPLC with the following parameters: solvent, methanol; mobile phase, 0.1% acetic acid and acetonitrile at a volume ratio of 25:75. Isocratic elution was performed with a flow rate of 0.8 mL/min, and the effluent was analyzed at a wavelength of 220 nm. Each sample (10 μL) was injected into an HPLC column for analysis.

### Data Nomenclature

In an electropherogram, the same uppercase letter was assigned to identical band types of different germplasms amplified using the same genomic SSR markers. The product of the allele was marked with an uppercase letter with the maximum marked as “A” followed by “B,” “C,” etc.; if only a single band was generated, the germplasm was recorded as homozygous. Different accessions with the same morphological trait were assigned different letters.

### Data Analyses

Genetic diversity was estimated using POPGENE version 1.32 (Yeh and Boyle, 1997), and it considered the expected heterozygosity (He), the observed number of alleles (Na), the expected number of alleles (Ne), Nei’s genetic diversity (I), and gene flow (*Nm*). The polymorphism information content (PIC) and major allele frequency (MAF) of each SSR were calculated using PowerMarker version 3.24 (Falush et al., 2007). The microsatellite bottleneck events were tested using 72 loci with eBOTTLENECK ver. 1.2.02. Three different models, namely the infinite allele model (IAM), stepwise mutation model (SMM), and two-phase model (TPM), were used to ascertain mutation-drift equilibrium (Cornuet and Luikart, 1996). A cluster analysis and genetic consistency assessment of different regions were also performed. A clustering map of different germplasm resources was drawn using MEGA version 7 (Kumar et al., 2016) following the unweighted pair group method with the arithmetic mean based on genetic distances. Population structure was assessed using a mixed model and a correlation model of allele variation frequency using STRUCTURE version 2.3.4 (Falush et al., 2007) and Structure Harvester version 6.0 (Earl and vonHoldt, 2012). The exact number of clusters (*K*) was evaluated by validating the *K*-value from 1 to 10 and a burn-in period of 5000 steps with 50,000 Markov chain Monte Carlo (MCMC) replications. The program Structure Harvester^2^ was run to assess the final K value for the STRUCTURE analysis, which was based on the plot of mean posterior probability [LnP(*D*)] values and the *ad hoc* Evanno’s △*K* statistics (Saha et al., 2019).

## Results

### Development of the SSRs Based on Genomic Data

A total of 92,409 SSR motifs were detected, and 63,699 (63.70%) pairs of SSR primers were developed. The most abundant SSR motifs were generally detected on chromosome 5 (12,099), whereas chromosome 10 showed the lowest number of SSR motifs (5277). The maximum ratio of SSR primers was 71.49% on chromosome 4, and the minimal ratio was 66.46% on chromosome 10. Chromosome 10 not only produced the lowest number of primers, but also had the lowest ratio; this was expected considering that chromosome 10 is a sex chromosome and contains less genetic information and variation than the other chromosomes (Table 1).

**TABLE 1:** Number of motifs and SSR primers.

Chromosome	Motif	Primer	Percentage
Chromosome 1	10854	7516	69.25%
Chromosome 2	10355	7087	68.44%
Chromosome 3	10528	7259	68.95%
Chromosome 4	9853	6828	69.30%
Chromosome 5	12099	8261	68.28%
Chromosome 6	8463	5701	67.36%
Chromosome 7	9913	6767	68.26%
Chromosome 8	8820	6195	70.24%
Chromosome 9	6547	4578	69.93%
Chromosome 10	5277	3506	66.44%

The dinucleotide SSR motifs were the most abundant type of repeats (82,859), followed by the trinucleotide, tetranucleotide, pentanucleotide, and hexanucleotide SSR motifs. According to the length of the genomic SSRs based on the number of repeat units (Table 2), the most abundant type of repeats was six, accounting for 42,305 (36.60%), followed by seven, five, eight, and nine repeat units. Only 6808 units presented more than 14 repeats (5.89%). The five, six, and seven repeat units accounted for 70.46% of the total repeat units, which could explain the predominant diversity of SSR repeat unit types. The number of primers developed based on each motif type was as follows: dinucleotide, 56,406 (68.07%); trinucleotide, 20,709 (71.94%); tetranucleotide, 1896 (62.10%); pentanucleotide, 356 (66.54%); and hexanucleotide, 289 (83.29%). Interestingly, the hexanucleotide motif type was the least common motif type, but it accounted for the highest percentage of the SSR primers developed, possibly caused by a complex of hexanucleotide type.

**TABLE 2:** Frequency of SSR motifs of genome-SSRs in hemp.

Motif length	Number of repeats									
	5	6	7	8	9	10	11	12	13	≥14
Di-		35,761	18,088	10,086	5,734	3,168	1,999	1,327	1,062	5,634
Tri-	15,372	5,882	2,482	1,382	889	577	411	334	289	1,168
Tetra-	2,366	527	114	28	9	4	1	1	1	2
Penta-	420	88	21	3	1	1				1
Hexa-	251	47	17	14	8	3	3	1		3

**FIGURE 1:**
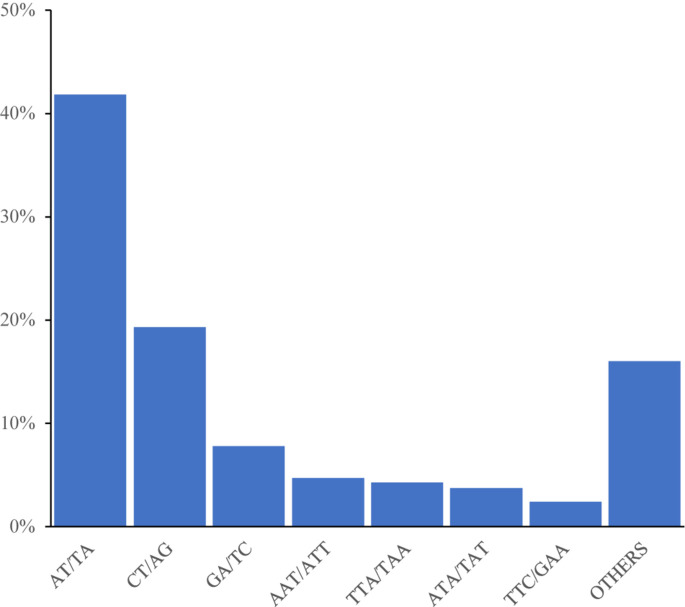
Frequency distribution of cannabis genome-SSRs based on motif numbers.

Among the 92,409 SSR motifs, 572 motif types were identified, namely di- (12), tri- (60), tetra- (122), penta- (187), and hexa- (191) types. The number of SSR motifs within each motif sequence type was as follows: di- (6904.9), tri- (496.3), tetra- (25), penta- (2.9), and hexa- (1.8) (Table 3). The di- type was the most abundant, and the hexa- type was the least abundant. The most abundant type of repeat motif was AT/TA, accounting for 48,353 of the repeats (41.84%), followed by CT/AG, GA/TC, and AAT/ATT; the other SSR motifs types (18,522) accounted for 16.03% of the repeats (Figure 1).

**TABLE 3:** Number of SSR motifs.

Type	Motif	SSR primers	Percentage
Di-	82,859	56,406	68.07%
Tri-	28,786	20,709	71.94%
Tetra-	3,053	1,896	62.10%
Penta-	535	356	66.54%
Hexa-	347	289	83.29%

According to the percentage of SSR primers on the 10 chromosomes, chromosome 5 produced the most abundant primers, and chromosome 10 produced the fewest. The results are consistent with the number of SSR motifs on each chromosome, and the percentages of the SSR primers were consistent with each other. A high number of motifs were associated with a high percentage of SSR primers across all chromosomes.

### Genomic SSRs and Phenotypic Markers

#### Overall Genetic Diversity

Eighty pairs of markers were randomly selected to evaluate the quality of the SSR markers across the 12 *Cannabis* varieties. A total of 11 pairs of markers from these 80 pairs failed to generate amplicons. Among the 69 pairs (86.25%) that generated amplicons, 59 (73.59%) showed polymorphisms that produced 72 loci. In addition, 13 pairs of primers produced 2 loci, and the remaining 10 markers had no polymorphisms. The 72 loci and the 3 phenotypic markers were then used to analyze the 199 germplasm resources and evaluate their population structure and genetic diversity.

PowerMarker and POPGENE analyses revealed that the Na ranged from 2 to 8 (average: 4.13), PIC ranged from 0.25 to 0.79 (average: 0.50), and I ranged from 0.50 to 1.78 (average: 1.01). The expected heterozygosity ranged from 0.28 to 0.81 (average: 0.56), Ne ranged from 1.38 to 5.32 (average: 2.50), and H ranged from 0.28 to 0.82 (average: 0.56) (Table 4). Considering two germplasm resources as a variety pair, the maximum genetic distance (1.0229) among the 19,701 pairs was observed between varieties 13 and 102, both from Gansu, China. The minimal genetic distance (0.2107) was observed between varieties 90 and 89, from Inner Mongolia and Heilongjiang, China, respectively.

**TABLE 4:** Characterization of 72 loci and 3 phenotypes.

Index	Mean	Min	Max	SD	CV
MAF	0.56	0.26	0.85	0.16	0.28
Na	4.13	2.00	8.00	1.25	0.30
H	0.56	0.28	0.82	0.13	0.24
PIC	0.50	0.25	0.79	0.13	0.27
He	0.56	0.28	0.81	0.13	0.24
Na	4.13	2.00	8.00	1.18	0.28
Ne	2.50	1.38	5.32	0.90	0.36
I	1.01	0.50	1.78	0.29	0.29

*MAF, major allele frequency; Na, observed number of allele; H, Nei’s gene diversity; He, expected heterozygosity; PIC, polymorphism information content; I, Shannon’s information index.*

#### Genetic Diversity Among Germplasms From Different Regions

Genetic diversity was also analyzed among 30 local cultivars and 109 wild *Cannabis* germplasm resources. The results show that the MAF, Na, Ne, I, H, PIC, and He were lower in local cultivars than in wild accessions; that is, the genetic diversity of wild accessions was higher than that of the domesticated accessions. The maximum Na value, 3.96, was recorded in wild accessions, and the minimum value, 3.4667, was recorded in the domesticated accessions. The difference between the maximum and minimum values of the other indexes was not very significant. We also analyzed 135 accessions from China and 64 accessions from abroad. The results show that Na was higher in Chinese accessions, and there were minimal differences in the other indexes between accessions from China and from other countries. In summary, domesticated accessions did not significantly differ from wild germplasms in China. The accessions from China did not significantly differ from foreign accessions although the Chinese accessions harbored more alleles than the foreign accessions.

The germplasms could be divided into nine regions, and the genetic diversity of germplasms among the nine regions was marginal (Table 5). The Na value was the highest for samples from Northeast China (3.79) and the lowest for samples from “other” (2.89). The largest value of MAF was found for samples from other, and the smallest was found for samples from Southwest China. Values of H, He, and PIC for the samples from the nine regions showed no differences; I was greatest in Northeast China and smallest in other; and Ne ranged from 2.18 (others) to 2.43 (Southwest China). In summary, there were no significant differences for the seven indexes among the samples from the nine regions. Samples from the other regions had the largest value of MAF and the lowest Na, H, PIC, I, and Ne.

**TABLE 5:** Genetic diversity of hemp with different geographic origins.

Region	MAF	Na	H	He	PIC	I	Ne
Southwest China	0.56	3.63	0.55	0.56	0.49	0.97	2.43
Middle China	0.58	3.61	0.54	0.55	0.47	0.95	2.38
Northeast China	0.58	3.79	0.55	0.55	0.49	0.97	2.41
Mid-east China	0.60	3.15	0.51	0.54	0.45	0.88	2.28
Northwest China	0.58	3.63	0.54	0.55	0.48	0.96	2.38
North China	0.59	2.93	0.51	0.56	0.45	0.87	2.27
Europe and America	0.56	3.64	0.55	0.56	0.49	0.97	2.45
Asia	0.58	3.51	0.54	0.55	0.48	0.95	2.40
Others	0.61	2.89	0.49	0.54	0.43	0.83	2.18

#### Genetic Distance and Genetic Consistency

**FIGURE 2:**
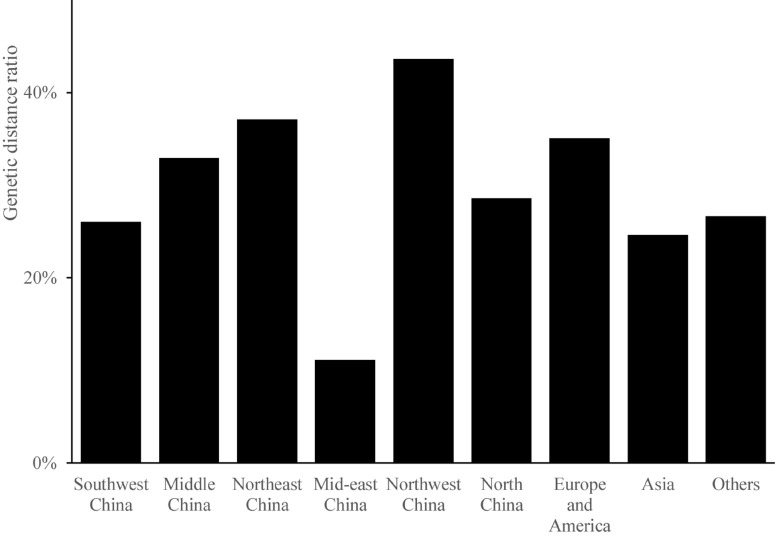
The genetic ratio of each region.

The magnitude of genetic distance reveals the genetic similarity among different groups. The genetic distance of germplasm resources ranged from 0.2107 to 1.0229. The average genetic distance was 0.4792, indicating a relatively low genetic variation among the 199 *Cannabis* germplasms. The pairwise genetic distances were usually 0.4–0.6 within each population (Figure 2). The percentage of germplasms showing a genetic distance greater than 0.5 was the highest in the case of samples from Northwest China (44%), indicating that the accessions in this region were less related to one another than those from other regions. Samples from mid-eastern China showed the lowest ratio of germplasms with a genetic distance greater than 0.5 (11%); the average ratio of the germplasms from the nine regions that showed a genetic distance greater than 0.5 was 30%. Further, the average ratio of all germplasms that showed a genetic distance greater than 0.5 except those from mid-eastern China were above 20%, indicating that *Cannabis* accessions from these regions were closely related.

The genetic distance between the samples from the various regions ranged from 0.0199 (between Northwest and Northeast China) to 0.1325 (between North China and other) (Table 6). The average genetic distance was 0.063, suggesting a relatively small genetic variation in accessions among the nine regions. Interestingly, there were two pairs with a genetic distance exceeding 0.100 among the 36 region pairs examined (between samples from North China and other, and between those from mid-eastern China and other). Moreover, the results indicate that samples from the region other did not show close relatedness to the samples from the remaining eight regions. The genetic consistency between the region pairs ranged from 0.8759 (between samples from North China and other) to 0.9803 (between samples from mid-eastern and Northwest China). The genetic consistency between samples from North China and other and between samples from mid-eastern China and other was below 0.900. In summary, genetic distances for all samples were below 0.2, and genetic consistency values were all above 0.8, demonstrating a close relatedness among the germplasms from the nine regions.

**TABLE 6:** Genetic distances and genetic consistency analysis.

Region	Southwest China	Middle China	Northeast China	Mid-east China	Northwest China	North China	Europe and America	Asia	Others
Southwest China	***	0.9671	0.9712	0.9391	0.965	0.9445	0.9611	0.9454	0.9099
Middle China	0.0335	***	0.9726	0.9401	0.974	0.9481	0.9497	0.9489	0.9221
Northeast China	0.0292	0.0277	***	0.939	0.9803	0.9479	0.9556	0.9526	0.917
Mid-east China	0.0629	0.0617	0.063	***	0.9383	0.9115	0.9238	0.9113	0.887
Northwest China	0.0356	0.0263	0.0199	0.0636	***	0.9447	0.9497	0.9505	0.9198
North China	0.0571	0.0533	0.0535	0.0927	0.0569	***	0.9208	0.9228	0.8759
Europe and America	0.0397	0.0516	0.0454	0.0792	0.0516	0.0825	***	0.963	0.9195
Asia	0.0562	0.0524	0.0486	0.0929	0.0507	0.0804	0.0377	***	0.9098
Others	0.0944	0.081	0.0867	0.12	0.0836	0.1325	0.084	0.0945	***

Lower left data of *** represent Nei’s genetic distance; Top right data of *** represent genetic consistency.

The average gene flow among all *Cannabis* accessions was 5.0974, and the mean fixation index (*Fst*) was 0.0468. High *Nm* and low *Fst* values indicate high levels of migration (Bossart and Pashley Prowell, 1998), which might be responsible for the small degree of variation observed within the 199 germplasm resources.

#### The Microsatellite Bottleneck Event

A bottleneck signature was detected for samples from all nine populations following their analysis using the IAM and TPM (with SMM = 30%) except for the samples from the region others (*P* = 0.0970), suggesting that a recent bottleneck event had occurred in all populations. However, all nine populations were found to show mutation-drift equilibrium following analysis using the SMM (Table 7). In the allele frequency distribution test, the samples from the regions mid-eastern China, North China, and other showed a shift in allele frequency distribution, presenting a shifted shape. The other populations showed no shift in allele distribution, maintaining a normal L-shape.

**TABLE 7:** Microsatellite bottleneck events.

Population	Sign test			Allele frequency distribution
	IAM	TPM	SMM	
Southwest China	**0.0000**	**0.0109**	0.3798	L-shaped
Middle China	**0.0000**	**0.0199**	0.2678	L-shaped
Northeast China	**0.0000**	**0.0184**	0.2150	L-shaped
Mid-east China	**0.0003**	**0.0395**	0.4586	Shifted mode
Northwest China	**0.0000**	**0.0244**	0.2895	L-shaped
North China	**0.0000**	**0.0025**	0.0892	Shifted mode
Europe and America	**0.0000**	**0.0005**	0.1595	L-shaped
Asia	**0.0000**	**0.0003**	0.5271	L-shaped
Others	**0.0078**	0.0970	0.3953	Shifted mode

*Sign test values were P-values, significant results for sign test were in bold.*

### Cluster Analysis

#### Germplasm Cluster Analysis

**FIGURE 3:**
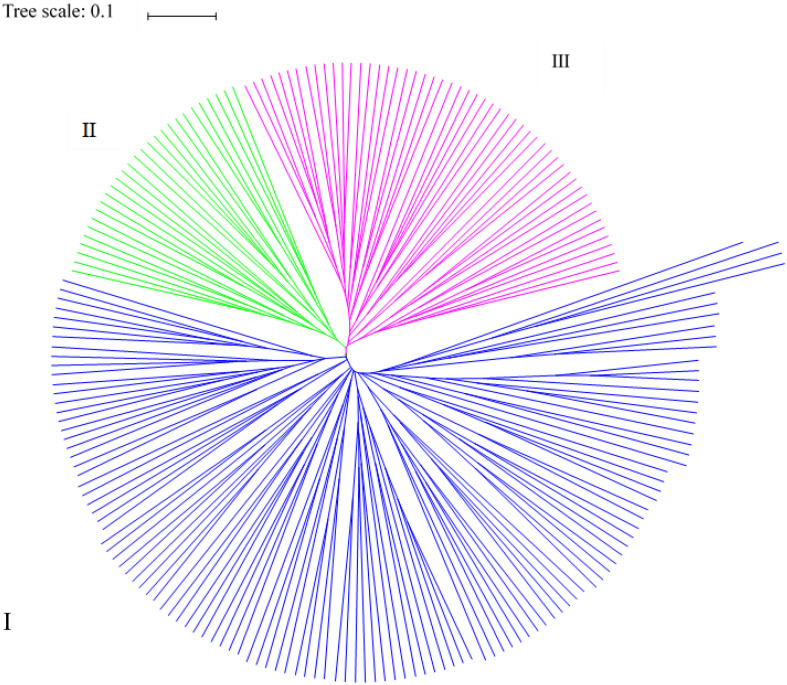
Cluster analysis results for the 199 hemp materials based on SSR and phenotypic markers.

Based on the genetic distances, all germplasm resources analyzed were divided into three classes using PowerMarker (Figure 3). The first class contained 119 germplasm resources, including those from Europe and America (21), Southwest China (22), Northeast China (23), Middle China (14), Northwest China (14), Asia (14), Mid-eastern China (6), North China (4), and other (1). The second class contained 28 germplasm resources from Southwest China (4), Middle China (6), Northeast China (6), Mid-eastern China (1), Northwest China (6), North China (1), Europe and America (1), and Asia (3) (and none from the group other). The third class consisted of 51 germplasm resources from Southwest China (5), Middle China (4), Northeast China (10), Mid-eastern China (3), Northwest China (4), North China (2), Europe and America (10), Asia (8), and others (5). Thus, the majority of germplasm resources from Southwest, Middle, and Mid-eastern China were clustered in the first class, and most of the germplasm resources from the others region were clustered in the third class. Germplasm resources from different regions were clustered into the same class in agreement with the genetic distance and genetic consistency values. The analysis indicates that the *Cannabis* germplasm resources have a similar genetic background.

#### Cluster Analysis of Different Regions

**FIGURE 4:**
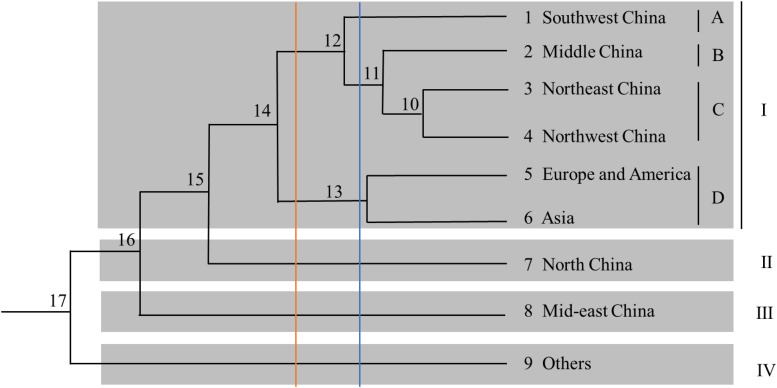
Dendrogram of the cluster analysis for the nine regions.

A phylogenetic tree based on Nei’s genetic distance was constructed (Figure 4). Arabic numbers are assigned to the nodes in the clustering map. At node 15, the nine regions are clustered into four groups (from top to bottom). Group I is further divided into four subgroups, namely A, B, C, and D, comprising six regions, whereas Groups II, III, and IV each contain only one region. Furthermore, Europe and America as well as Asia are clustered in Group I, indicating that the samples from the first two regions are closely related to those from almost all Chinese regions. At node 14, the nine regions are separated into five groups; subgroup D is integrated into Group II, and Groups III, IV, and V contain only one region each. At node 12, the nine regions are categorized into five groups (from top to bottom): Group I contains four regions; Group II contains two regions; and Groups III, IV, and V contain one region each. These results reveal that the germplasm resources from China are not clustered within a certain group and that there is no direct correlation between region and affiliation. Subgroup D is categorized into one independent group at nodes 11, 12, and 14. In general, the analysis of region clusters based on genetic distance and diversity indexes reflects the geographical origin of the germplasms.

### Population Structure of Cannabis Germplasms

**FIGURE 5:**
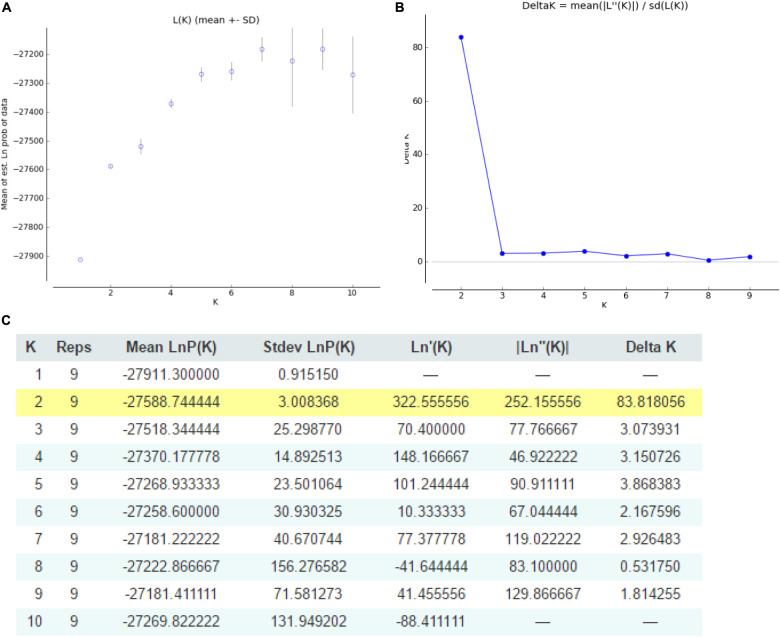
Graphical representation of the population structure. **(A)** *K*-values for different numbers of populations assumed (*K*) in the STRUCTURE analysis. **(B)** The median and variation of the estimated probability value for each *K*-value. **(C)** Evanno table output.

**FIGURE 6:**
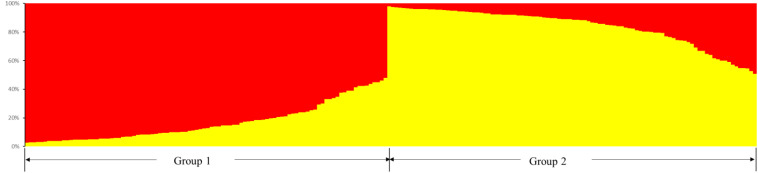
Population structure of the 199 hemp germplasm resources based on the SSR and the phenotypic markers.

The structure of *Cannabis* germplasm resource genotypes was analyzed based on the likelihood of data [LnP(*D*)]. The germplasm resources were randomly integrated into groups (*K*) to assess the variant frequency of each group, and the individual germplasms were reintegrated into groups based on the estimated frequencies (Evanno et al., 2005). The number of subgroups varies with the LnP(*D*) values. The curve occurs between *K* = 1 and *K* = 2, and all the values are at their maximum when *K* = 2 (Figure 5), i.e., when the germplasm resources are divided into two subgroups (Figure 6), including 99 and 100 genotypes, which accounts for 49.60% and 50.40% of the germplasm resources, respectively. The first subgroup includes accessions from Southwest China (18), Europe and America (27), Asia (22), and all the germplasms in the set others (6). The second subgroup includes those from Southwest China (13), Middle China (18), Northeast China (28), and Northwest China (24). The structure of each subgroup varies except that accessions from the others region are not included in the second subgroup. The first subgroup is generally composed of accessions from abroad, and the accessions in the second subgroup are all from China. The cluster analysis reveals that the genotypes in both subgroups are variable, but almost all genotypes show the same general trend.

## Discussion

Various types of molecular markers are used to assess the genetic diversity of *Cannabis*, such as ribosomal DNA, inter-simple sequence repeats (ISSRs), sequence characterized amplified regions, random amplified polymorphic DNA (RAPD), and amplified fragment length polymorphism (AFLP). In one study, 10 pairs of AFLP primers are validated to identify illicit *Cannabis* cultivars (Datwyler and Weiblen, 2006). Other studies use RAPD markers to identify prefloral hemp (Shao et al., 2003) and the RAPD marker OPA8 that generates specific 400-bp bands in male but not in female plants (Mandolino et al., 1999). A genetic diversity analysis of 27 Chinese hemp cultivars using ISSR markers reveals that the accessions can be classified into five categories (Zhang et al., 2014).

SSR markers are based on microsatellites and are considered the most efficient and abundant molecular markers with a high ratio of genome coverage. They are highly reproducible and can be used to study codominant inheritance (Chen et al., 2019). Moreover, SSR polymorphisms are employed to identify and characterize germplasm resources in terms of affiliation (Tuler et al., 2015), and the development and characterization of genomic SSRs in hemp are important to enable genetic research and marker-assisted selection. Because SSR markers have a higher number of polymorphisms than other molecular markers, they are popular and ideal for analyzing population structure and genetic diversity and identifying fiber crop varieties (Zhang et al., 2015a, b; Saha et al., 2017, 2019). In previous research, EST-SSR development was conducted, and 4577 potential SSR motifs were identified for *Cannabis* (Gao et al., 2014).

Genomic SSRs have the advantages of a higher number of polymorphisms and higher stability than EST-SSR markers (Ding et al., 2017). Thus, SSR markers in *Cannabis* can be valuable in future research. In the present study, 92,409 SSR motifs are detected in the *Cannabis* genome, from which 63,707 pairs of SSR primers are developed, meaning 63.7% of motifs with developed primers. The most abundant sequence motif is of the dinucleotide type (56,406), and the most abundant repeat motif is AT/TA (41.84%). These results differ from those of previous studies, which report that the trinucleotide AAG/CTT and dinucleotide AG/CT are the most common types in EST-SSR (Alghanim and Almirall, 2003; Gao et al., 2014). The most abundant motifs are detected on chromosome 5, suggesting this chromosome may have abundant genetic information and several potentially modified loci. Among the 80 pairs of genomic SSRs, 59 have validated polymorphisms. This information is valuable for the development of *Cannabis* fingerprints to aid in cultivar identification. Knowledge of the genetic diversity and population structure of crop germplasm resources could accelerate genetic research and the development of new plant varieties. Thus, the results obtained herein may also aid in conserving and utilizing specific high-quality germplasm resources.

*Cannabis* shows higher genetic diversity than annual wind-pollinated and gravity-dispersed weedy plants (Lynch et al., 2016). Historically, *Cannabis* germplasm resources around the world are limited. In the 1970s, because of the hallucinogenic effects of *Cannabis*, the United States prohibited *Cannabis* planting, and other countries followed. However, in the 1990s, scientists determined that *Cannabis* had positive treatment effects on various diseases, and *Cannabis* farming was accepted worldwide (Chandra et al., 2017). However, the ban on *Cannabis* farming for several years affected global germplasm resource collection and our knowledge of its genetic structure and diversity.

In a previous study, the genetic diversity and DNA fingerprinting of jute was analyzed by 28 pairs of SSR primers, a total of 184 polymorphic loci were identified, and the DNA fingerprinting of 58 jute accessions was based on SSR markers (Zhang et al., 2015a). The genetic differentiation and population structure of 93 fiber flax accessions were evaluated based on genome-wide regulatory gene-derived SSRs and all accessions separated into two subpopulations: Indian and global (Saha et al., 2019). In the present study, PIC values > 0.5 indicate that the locus is highly informative, 0.25 < PIC < 0.5 represent moderate polymorphisms, and PIC values < 0.25 mean a low rate of polymorphism (Jensen et al., 2011). Because PIC ranges from 0.25 to 0.78 (mean: 0.50), *Cannabis* germplasm resources are considered to have a high degree of polymorphism. The values are lower than those of the Kenyan common bean (*Phaseolus vulgaris* L.) (Valentini et al., 2018), Indian garlic (*Allium sativum* L.) (Kumar et al., 2019), and ramie (*Boehmeria nivea* L.) (Feng et al., 2018) and higher than those of tea plants (*Camellia sinensis* L.) (Ori et al., 2017), corn (*Zea mays* L.) (Zhang et al., 2017), and sesame (*Sesamum indicum* L.) (Yue et al., 2012). In addition, the PIC values of the domesticated, wild, and Chinese and foreign accessions range from 0.25 to 0.50, indicating that the germplasm resources in these regions have moderate polymorphism, and the results are consistent among the nine populations. The He of the accessions from the nine regions is around 0.55; when combined with the MAF (0.6), this indicates that the alleles are uniformly distributed across the populations. The I is around 0.9, which reveals that *Cannabis* has a highly stable genetic structure. The values of I, Ne, MAF, He, and H among the 199 germplasm resources from the nine regions range between 0.93 and 1.01, 2.35 and 2.50, 0.58 and 0.56, 0.55 and 0.56, and 0.53 and 0.56, respectively. The observed and effective numbers of alleles differ among the populations except for the samples from the other region; those from the other regions have an uneven distribution of alleles. The accessions show a stable genetic structure and moderate genetic diversity. Compared with the He value of 0.49 and I value of 0.32 reported previously (Gao et al., 2014), the accessions in the present study show high genetic diversity. The similarity between the populations analyzed with regard to genetic diversity is confirmed to be a result of a genetic bottleneck event. Following analysis using the IAM, the nine populations do not show mutation-drift equilibrium, suggesting a substantial erosion of genetic diversity among the populations.

In the present study, the germplasm resources are clustered in three groups, and each group includes samples from most, if not all, of the nine regions. In a previous report, 115 *Cannabis* accessions are divided into four groups, and the genetic diversity between Northern China and Europe is higher than that between groups containing accessions from China only (Gao et al., 2014). Thus, *Cannabis* germplasms cannot always be clearly distinguished based on geography although geographic origin could aid in domesticating certain varieties and introducing new ones. LnP(*D*) varies with the number of subgroups, but there is no obvious inflection point in the curve. The population structure analysis and mathematical model application divide the 199 germplasm resources into two subgroups in a population with a single structure.

*Cannabis* is believed to have originated in China, central Asia, and the northwest Himalayas (Hillig, 2005). The genetic structure of marijuana and hemp are significantly different (Sawler et al., 2015). Genetic differentiation varies with genetic frequency as well as with genetic drift and heterozygosity. The fixation index expresses the degree of genetic differentiation in the population at four levels: little (0 < *Fst* < 0.05), moderate (0.05 < *Fst* < 0.15), large genetic (when 0.15 < *Fst* < 0.25), and very large genetic differentiation (0.25 < *Fst* < 1) (Wright, 1965). The 199 germplasm resources show little genetic differentiation with an *Fst* value of 0.0468. Gene flow is a vital index to detect genetic leakage and assess genetic differentiation. When the gene flow (*Nm*) is >1, the population is able to efficiently prevent the genetic differentiation caused by genetic drift; otherwise, genetic differentiation is inevitable (Wright, 1965; Golenberg, 1987). Gene flow in the present study is 5.0974, indicating that *Cannabis* germplasm resources around the world frequently undergo gene exchange, which efficiently reduces the genetic differentiation caused by genetic drift. Accordingly, the genetic distance among the nine regions is low, which is consistent with the results of a previous review (Chandra et al., 2017). However, this genetic distance shows large variation. There is no difference among the cultivars and wild *Cannabis* varieties in China or between the Chinese and foreign *Cannabis* germplasms, but this result is not consistent with the findings of previous reports (Lynch et al., 2016). The genetic consistency of the germplasms analyzed herein is higher than 0.9, suggesting that the accessions from all nine regions have a relatively high kinship.

Because CBD and THC are unique to *Cannabis*, they are used as phenotypic markers in the present study (Piomelli and Russo, 2016). CBD and THC show therapeutic potential for many diseases (Galasso et al., 2016). To classify *Cannabis* varieties, the most accepted standard is a level of 0.3% for THC and 0.5% for CBD. When the THC content is >0.3% and the CBD content is <0.5%, the *Cannabis* is considered to be of the drug type; when the THC content is <0.3% and the CBD content is <0.5%, the *Cannabis* is of the fiber type; and when the THC content is <0.3% and the CBD content is >0.5%, it is considered to be of the medicinal type (Small and Beckstend, 1973; Small and Cronquist, 1976). In the present study, the contents of CBD in the 199 *Cannabis* accessions ranges from 0.00% to 0.93% (average: 0.15%), and the THC contents range from 0.00% to 0.75% (average: 0.18%). The variable coefficients are large for the CBD and THC contents at 108% and 82.6%, respectively. However, based on their THC and CBD contents, 33 accessions are of the drug type, 135 of the fiber type, 7 of the medicinal type, and 24 have inadequate data for classification.

Overall, the genetic diversity and population structure analyses presented herein provide a basis for the further investigation of *Cannabis* species by quantitative trait loci mapping, association analysis, and molecular-assisted breeding. Our findings can also further the exchange of *Cannabis* germplasms among different areas in China and the introduction of new *Cannabis* varieties from abroad.

## Data Availability Statement

All datasets generated for this study are included in the article/Supplementary Material.

## Author Contributions

LZ, DL, and DP designed the experiments. SH, JL, and AC provided the research materials. AB and CuZ performed polyacrylamide gel electrophoresis and data analysis. GP, LC, ChZ, and HT measured the contents of CBD, THC, and CBDA. JZ and JY wrote the manuscript.

## Conflict of Interest

The authors declare that the research was conducted in the absence of any commercial or financial relationships that could be construed as a potential conflict of interest.

## Footnotes

1 http://pgrc.ipk-gatersleben.de/misa/

2 http://taylor0.biology.ucla.edu/structureHarvester/#
