# Supplementary material for: Genetic Diversity and Population Structure of Cannabis Based on the Genome-Wide Development of Simple Sequence Repeat Markers
Source: Front Genet. 2020 Sep 11;11:958. doi: 10.3389/fgene.2020.00958 (PMC7518120; doi:10.3389/fgene.2020.00958)
Supplement: Supplementary file 1 [file Data_Sheet_1.zip › Data Sheet 1/Supplementary Material/Supplementary Figures.docx]

**Fig.S1. Percentage of SSR primers associated with 10 chromosomes**

**Fig S2. Frequency of SSRs of different motif repeats**
